# Genetic, Diversity, and Muscle Quality Among Red and Green Color Morphs of Asian Swimming Crab (*Charybdis japonica*): Implications for Accurate Species Recognition and Sustainable Management

**DOI:** 10.3390/foods14142516

**Published:** 2025-07-18

**Authors:** Bingqian Zhang, Yuhang He, Maninder Meenu, Ying Liu, Yusheng Jiang

**Affiliations:** 1 Sanya Tropical Fisheries Research Institute, Sanya 572018, China; yuhanghe821@163.com; 2 Dalian Key Laboratory of Breeding, Reproduction and Aquaculture of Crustaceans, Dalian Ocean University, Dalian 116023, China; zhangbingqian1999@icloud.com; 3 Dalian Jinshiwan Laboratory, Dalian 116014, China; 4 Department of Biosystems Engineering, Zhejiang University, 866 Yuhangtang Road, Hangzhou 310058, China; meenu_maninder@yahoo.com (M.M.); liuyingzju@zju.edu.cn (Y.L.)

**Keywords:** Asian swimming crab *Charybdis japonica*, body color, genetic, phenotypic diversity, muscle quality

## Abstract

In this study, two color morphs (red and green) of Asian swimming crab (*Charybdis japonica*) commonly distributed in the China Sea area were analyzed for their L*a*b* values, carapace and inner membrane histology, morphological characteristics, mitochondrial DNA sequences, muscle texture, and amino acid composition. The results showed that compared with the green morph group, the red morph group exhibits higher aggregation of melanocytes and fewer pigment cells in the inner membrane. In addition, L* and b* of the carapace, and L* values of the inner membrane were lower in red morph group. Both populations of *C. japonica* also exhibit significant differences in their morphological parameters, including carapace length, body weight, and pincer width. However, the coefficient of variation for these morphological parameters did not correspond to the subspecies level. The mitochondrial DNA analysis also revealed sequence identity of COI (98.96%) and ITS-1 (99.71%) genes in both groups, supporting them to belong to the same species. Both groups also presented significant differences in their muscle texture characteristics, including adhesiveness, springiness, and gumminess, but no significant differences were observed in the muscle amino acid composition. Overall, red and green morphs of *C. japonica* show differences in their body color, morphological characteristics, and muscle quality, but still belong to the same species.

## 1. Introduction

The color of crustacean body serves several fundamental biological functions, including communication, reproduction, thermoregulation, and defense against predators [1,2]. Additionally, changes in body color play a critical role in improving environmental adaptability and regulating reproductive behavior, which is ultimately linked to the survival and ecological success of crustaceans [3]. Beyond inter-species differences in body color among crustaceans, substantial intra-species variations also exist due to body size, sex, molting stages, and geographic distribution [4,5]. The quality and economic value of crustaceans are often associated with their body color. Thus, their body color is frequently selected and utilized as an important economic trait in artificial breeding and aquaculture. Previously, studies on the body color of crabs have primarily focused on the characteristics and metabolism of pigments [6,7]. McNamara et al. conducted a comprehensive review of molecular endocrinology of pigment cell translocation in crustaceans, emphasizing their dynamic response to environmental stimuli such as light intensity and substrate background [8]. However, studies on the eco-genetic mechanisms underlying body color diversity in natural populations and their association with quality traits are scarce, and species identification is often misjudged due to morphological plasticity. Accordingly, a hypothesis was proposed suggesting that two color morphs of Asian swimming crab (*Charybdis japonica*) are genetically and environmentally regulated, and body color is functionally correlated with muscle textural traits. Therefore, a classification framework integrating morphological and molecular data is required for two color morphs of *C. japonica*.

*C. japonica* is a commercially valuable species widely distributed in China, Japan, the Korean Peninsula, and Southeast Asian countries [9]. *C. japonica* is popular among consumers due to its unique flavor and high nutritional value. *C. japonica* also exhibits strong environmental adaptability, which makes it a promising species in aquaculture [10,11]. However, the catch volume and individual size of *C. japonica* have been declining annually due to the over-exploitation of natural resources and the degradation of coastal ecosystems [12]. Consequently, in recent years, the breeding technology, physiological status, genetic structure, and fishery resource of *C. japonica* have garnered significant attention from the scientific community [13,14,15]. In natural waters, two distinct color morphs of *C. japonica* are commonly observed, one exhibiting an overall purple-red color, and the other displaying bluish (or yellowish) gray cephalothorax and cheliped dorsum and a completely white ventral surface. To date, there have been few reports on the color differentiation phenomenon in *C. japonica*, which causes consumers to perceive both color morphs differently. Therefore, it is essential to investigate differences in their biological characteristics and explore the underlying mechanisms responsible for color differentiation.

Consumers are especially curious whether these two-color morphs of *C. japonica* belong to the same species. In general, morphological characteristics serve as key indicators for species classification and resource identification, with their complexity and variability providing reliable evidence for identifying and distinguishing species [16]. The morphological study has always been preferred for species classification and phylogenetic research due to its intuitive nature and ease of operation. Previously, morphological studies of sawtoothed crabs (*Scylla serrata*) have revealed significant differences between farmed and wild crabs, which are related to their ability to defend against predators [17]. However, the external morphological features of crustaceans can exhibit a certain degree of plasticity in response to changes in environmental factors [18]. Thus, identification based on morphological characteristics alone can lead to misinterpretation. The application of modern molecular biotechnology techniques overcomes the shortcomings of morphological methods and complements their results. Mitochondrial DNA (mtDNA) is widely used to study genetic diversity and phylogenetic relationships in crustaceans due to its maternal inheritance, high mutation rate, and lack of recombination [19]. The mtDNA markers have been extensively applied in crustaceans, providing valuable insights into their evolutionary history and genetic differentiation. Previously, genetic analysis based on the mitochondrial cytochrome oxidase subunit I (COI) gene sequence was conducted for the mud crab (*Scylla paramamosain*), revealing low levels of genetic differentiation among mud crabs populations in different coastal regions of China [20]. Ren et al. investigated the genetic diversity and population structure of blue swimming crab (*Portunus pelagicus*) using mtDNA molecular markers and reported high levels of gene flow along the distribution areas of China [21]. Therefore, the accuracy of crustacean species identification can be improved by comprehensive morphological and mitochondrial gene data analysis. The muscle of *C. japonica*, the primary edible component, is also a key focus for consumers. The body color is typically associated with the quantity and type of carotenoids, and their accumulation can also lead to changes in muscle quality [22,23]. Therefore, it is important to investigate differences in the textural characteristics and flavor profiles of muscles from both color variants of *C. japonica*.

Thus, this study compared the morphological parameters, mitochondrial COI and ITS gene fragment sequences, muscle textural characteristics, and amino acid composition of two color morphs (red and green) of *C. japonica*. The results of present study will provide a theoretical basis for further elucidating the underlying mechanisms responsible for color differentiation in *C. japonica* and also provide fundamental knowledge for establishing selective breeding techniques for *C. japonica* based on color.

## 2. Materials and Methods

### 2.1. C. japonica Collection

In this study, 188 wild adult *C. japonica* were collected from the coast of Dalian, Liaoning province, China. These live crabs were transferred to the Dalian Key Laboratory of Breeding, Reproduction and Aquaculture of Crustaceans, Dalian Ocean University, for further analysis. Before the experiment, healthy individuals exhibiting distinctly different body colors were selected based on visual observation and subsequently categorized into two groups according to their body color. A total of 136 *C. japonica* were finally selected, including 65 “red morphs” and 71 “green morphs”. These crabs were temporarily stored at a density of 6–7 per tank in 20 plastic tanks (length × width × height = 70.5 cm × 49 cm × 39 cm) containing sand-filtered seawater at 20 °C with continuous aeration and salinity at 30, and light cycles set as natural light. During the temporary holding period, crabs were fed to minimize the influence of additional factors on the subsequent experiments. In addition, many shelters and sand were provided in the tanks to reduce aggressive interactions between individuals and alleviate stress responses.

### 2.2. Determination of Morphological Parameters

**Figure 1:**
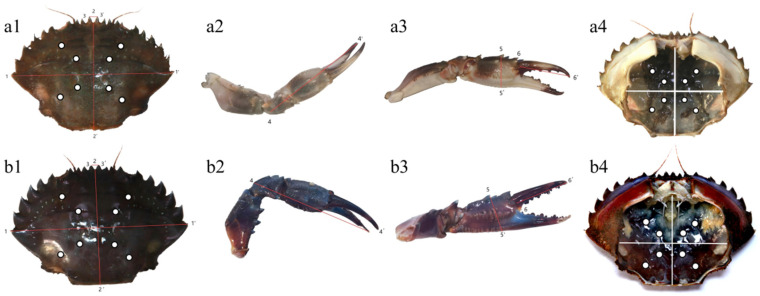
Morphological measurement landmark points of two color morphs of *Charybdis japonica*, the landmark points of green morph (**a**) and red morph (**b**). The landmark point of carapace (**a1**, **b1**), carapace length (CL): 1-1′, carapace width (CW): 2-2′, first obital margin width (FOMW): 3-3′. The landmark point of left chelipeds (**a2**, **a3**, **b2**, **b3**), fixed finger length of claw (FFLC): 4-4′, meropodit length of claw (MLC): 5-5′, pincer width (PW): 6-6′. The white points represent the measurement points of Lab* values for the carapace and inner membrane (**a1**, **a4**, **b1**, **b4**).

The body weight (BW) was measured using an electronic balance after removing moisture from *C. japonica*. The carapace and chelipeds were measured using a vernier caliper. The carapace length (CL), carapace width (CW), first obital margin width (FOMW), fixed finger length of claw (FFLC), meropodit length of the claw (MLC), and pincer width (PW) were recorded according to the landmark points as shown in Figure 1. Subsequently, the coloration and sex of the specimen were also recorded. In contrast, the shell thickness (ST) of *C. japonica* was measured after dissection. To mitigate the influence of size variation on morphological traits in *C. japonica*, all measured morphometric parameters were converted into ratios relative to the carapace width. Based on the ANOVA results, coefficient of difference (CD) calculations were conducted for the morphometric ratios that showed significant differences between the two body color morphs of *C. japonica*. The coefficient of difference (CD) was calculated according to a formula as follows:

CD = |M_1_ − M_2_|/(S_1_ + S_2_)

M denotes the average, while S indicates the standard deviation of the morphometric trait for each morph. The CD is ≥1.28 presents a significant difference between the two color morphs at subspecies level or above.

### 2.3. DNA Extraction and Sequencing

*C. japonica* (*n* = 10 per group) were randomly selected from each group presenting different body colors. For each sample, 0.1 g of appendage muscle tissue was finely minced and incubated with 500 μL of DNA lysis buffer (consisting of 10 mmol/L Tris-HCl, pH 8.0; 50 mmol/L EDTA, pH 8.0; and 0.5% SDS). After thorough mixing, proteinase K was added at a concentration of 20 μg/mL, followed by incubation at 55 °C until clear. The DNA was extracted using the phenol-chloroform method and stored at −20 °C for further analysis. The primers specific for the mitochondrial COI and ITS-1 gene fragments of *C. japonica* were synthesized by Sangon Biotech Co., Ltd. (Shanghai, China) based on sequences previously reported in the literature. The primer sequences for COI were GGTCAACAAATCATAAAGATATTGG (forward) and TAAACTTCAGGGTGACCAAAAAATCA (reverse). For ITS-1, the forward primer sequence was CACACCGCCCGTCGCTACTA, and the reverse primer sequence was ATTTAGCTGCGGTCTTCATC. The PCR reaction mixture (25 μL) was composed of DNA template (30 ng), 10 × PCR Buffer (2.5 μL), MgCl_2_ (2 μL), dNTPs (0.5 μL), Taq DNA polymerase (0.13 μL), 0.5 μL of each primer (10 μM), and 17.87 μL of sterile ultrapure water. The PCR was conducted at an initial denaturation temperature of 94 °C for 3 min, followed by 35 cycles at 94 °C for 30 s, 55 °C for 1 min, and 72 °C for 1 min, and final extension at 72 °C for 5 min. The amplified products were visualized on 1% agarose gel and subsequently sequenced by Sangon Biotech Co., Ltd. (Shanghai, China).

All acquired sequences were edited using BioEdit software (V7.0.9.0), complemented by manual verification to ensure accuracy. The sequence alignment was conducted with ClustalW2 (V2.1) to determine sequence lengths. The base composition, variable sites, parsimony-informative sites, and genetic distances were calculated using MEGA5.0 software. The phylogenetic tree among both color variants of *C. japonica* and related outgroup species was constructed using both Neighbor-Joining (N-J) and UPGMA methods. For analyzing average nucleotide differences, crustacean sequences (specifically from *Charybdis feriatus*, *Charybdis acuta*, *Portunus pelagicus*, *Portunus trituberculatus*, *Scylla serrata*, *Eriocheir sinensis*, *Callinectes saqidus*, and *Macrobrachium nipponense*) from GenBank were used as reference. The detailed information on accession numbers and fragment lengths of these outgroup sequences is provided in Table 1.

### 2.4. Determination of Body Color and Histological Examination

The images of inner membrane and carapace of two color morphs were captured using a Color Cue 2 colorimeter (PANTONE, Carlstadt, NJ, USA) to obtain the L*, a*, and b* values. The measurement points of L*, a*, and b* values on carapace and inner membrane of *C. japonica* were shown in Figure 1. Six crabs from both groups were selected, and eight relatively smooth measurement points were chosen on each crab. The results of L*, a*, and b* values were presented as averages to reduce color variability. For histological examination, the samples were fixed in 10% neutral-buffered formalin for 48 h, followed by dehydration in a graded series of ethanol and paraffin embedding. The samples were sectioned into thin slices (5 μm) using a rotary microtome and stained with hematoxylin-eosin (H&E) for morphological observation. The histopathological evaluation was conducted using a Nikon Eclipse H600L microscope equipped with a digital imaging system (NIS-Elements BR, Nikon Instruments, Tokyo, Japan) to observe structural integrity and pathological alterations of the epidermis and chitin layer.

### 2.5. Analysis of Muscle Texture and Amino Acid Content

To measure the muscle texture, three individuals of two color morphs of C. *japonica* were randomly selected and cut along the cheliceps joints with dissecting scissors to avoid muscle damage caused by compression. Then, the muscle was cut at the junction with the shell using a scalpel to obtain the cheliped muscle. The resultant muscle samples were cut into cubes (length × width × height = 10 mm × 10 mm × 10 mm) to observe hardness (N), adhesiveness (N.mm), cohesiveness (adhesiveness), springiness (mm), gumminess (N), and chewiness (mJ) using texture analyzer (TMS-Pro, FTC, Columbia, MD, USA) by employing a previously reported method [24,25]. For textural analysis, parameters were set as follows: cylindrical P20 probe, trigger point load of 10 g, compression target value of 5 mm, test speed of 2 mm/s, cycle times 2 m. The average value of two cheliped muscle as a result of the individual’s muscle texture. Each sample was measured six times in parallel, and the average value was taken.

The amino acid composition of the muscle samples was explored according to a previously reported method [26]. Three individuals from each group were randomly selected and cut along the walking leg joints with dissecting scissors. After removing exoskeleton fragments and connective tissues, the muscle sample (0.2 g) was mixed with 10 mL of HCl, followed by hydrolysis for hours at 110 °C in an electric thermostatic drying oven. After cooling, the hydrolyzed sample was diluted to 20 mL with distilled water. Then, the hydrolyzed sample (100 μL) was placed in a vacuum drying oven at 60 °C for 2 h for complete evaporation of solvent. Subsequently, nitrogen gas was introduced to eliminate any residual solvent traces. After adding derivatization reagent (ethanol:phenylisothiocyanate:water:triethylamine = 7:1:1:1), the mixture was allowed to react for 30 min at room temperature. Then, the solution was thoroughly mixed with 0.45 mL of mobile phase A (acetonitrile solution). The mixture was filtered through a 0.45 µm organic solvent-resistant membrane filter before being injected into high-performance liquid chromatography (HPLC) system (Agilent 1260) equipped with a C18 column (SHISEIDO, 4.6 mm × 250 mm × 5 μm) at 40 °C, and the detector was set at 254 nm. The amino acid content in the sample was calculated using the following formula:

W = m × (C − C_0_) × V × N

where: W: Content of target amino acid in the sample (mg/kg); C: Concentration of target amino acid in the sample solution (mg/L); C_0_: Concentration of target amino acid in the control (mg/L); V: Final volume of sample solution (mL); N: Dilution factor; m: Mass of the sample taken (g).

### 2.6. Statistical Analysis

The data were presented as mean ± SD. The significant differences (*p* < 0.05) in various parameters among both groups were analyzed using a *t*-test. The normal distribution and homogeneity of variance were applied to all the data. All statistical analysis was performed using SPSS 23.0 software (IBM Corp., Armonk, NY, USA).

## 3. Results

### 3.1. Body Color and Histological Observations

**Figure 2:**
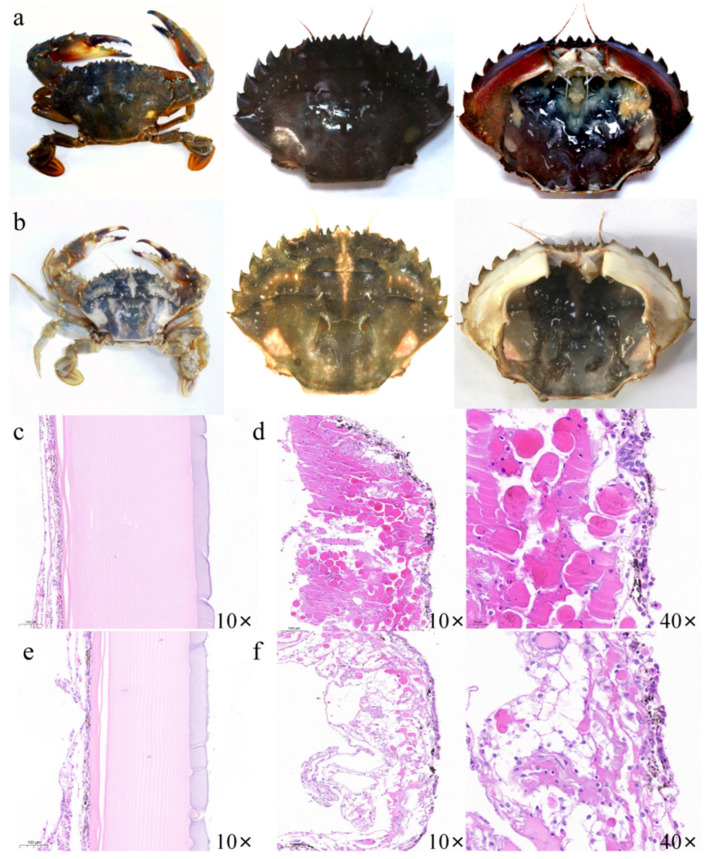
Observation on shell and inner membrane of two color morphs of *C. japonica*. (**a**): red morph; (**b**): green morph; (**c**): shell section of red morph; (**d**): inner membrane section of red morph at two observation multiples (10× and 40×); (**e**): shell section of green morph; (**f**): inner membrane section of green morph at two observation multiples (10× and 40×).

The histological observations revealed the distribution of pigment cells (melanocytes) in both the shell and inner membrane of crabs. The red morph group presented a thicker epidermis and a more extensive distribution of pigment cells compared to the green morph group. In case of green morph group, a distinct color partition was visually observed at the junction between shell and inner membrane; thus, this particular area was also selected for observation in the red morph group. In the red morph group, melanocytes were found to be aggregated, whereas in the green morph group, they were sparse and more evenly distributed. In the green morph group, the surface of the inner membrane exhibits several pigment cells combined with pigment proteins to form pigment deposits, while the surface of the inner membrane in the red morph group exhibits fewer evenly distributed pigment cells. The red morph group also exhibits distinct red pigment cells and melanocytes. These red pigment cells were located beneath the melanocytes. In contrast, the green morph group only had a few melanocytes between the epidermis and dermis, which is involved in the continuous secretion of melanin granules into epidermis (Figure 2).

**Figure 3:**
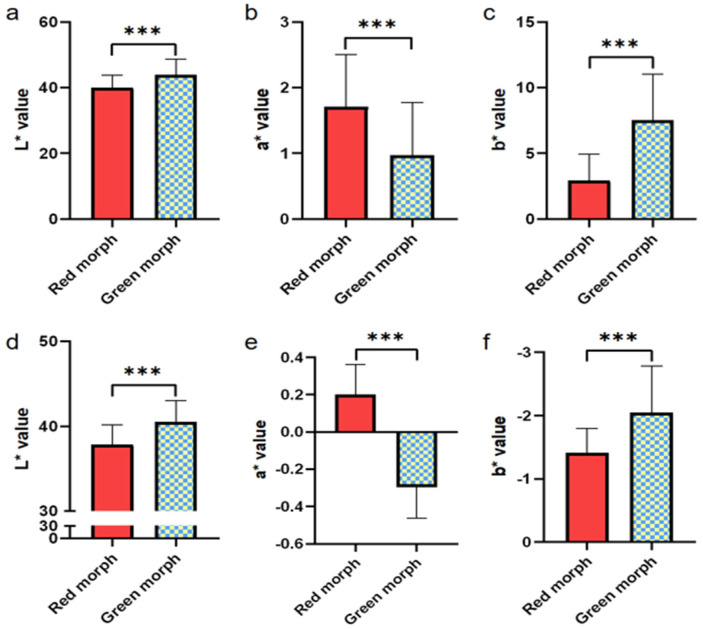
The Lab* value difference of carapace (**a**–**c**) and inner membrane (**d**–**f**) of two color morphs of *C. japonica*. Vertical bars represent the mean ± SD. *** means significant difference (*p* < 0.0001).

The variation in color values (L*, a*, b*) of carapace and inner membrane of both groups is shown in Figure 3. The L* and b* values of carapace in red morph group were significantly lower (*p* < 0.05) compared to those in the green morph group. In contrast, the a* value of red morph carapace was significantly higher (*p* < 0.05) compared to green morph group. The Lab values of inner membrane showed a similar trend to those observed in the carapace. Compared to the green morph group, the red morph group exhibited significantly lower L* and b* values in the inner membrane (*p* < 0.05), whereas the a* value was significantly higher (*p* < 0.05).

### 3.2. Morphological Characteristics of C. japonica

In the present study, *C. japonica* samples were collected from the Heishijiao Sea area of Dalian, and the data obtained follow a normal distribution (Figure S1). The phenotypic statistical scale of *C. japonica* from both groups is shown in Table 2. In male *C. japonica*, the red and green morph groups presented significant differences (*p* < 0.05) in morphological parameters, including CL, CW, BW, FOMW, FFLC, MLC, PW, and ST. However, no significant differences (*p* > 0.05) were observed in these morphological parameters between the two color morph groups of female *C. japonica*.

The ratio of each morphological parameter to the CW was used to reduce the impact of individual size differences on the recorded data of morphological data, as shown in Table 3. Compared to the males in green morph group, the males in red morph group showed significantly lower (*p* < 0.05) CL/CW, whereas PW/CW and BW/CW were significantly increased (*p* < 0.05). However, no significant differences (*p* > 0.05) were observed in these morphological parameters between females of both groups. Subsequently, a coefficient of variation was analyzed in case of these three morphological parameters between male crabs from both groups (Table 4). The coefficient of variation for these three morphological parameters was below 1.28, indicating that observed morphological differences stem from variations among distinct geographic populations and do not warrant differentiation at the subspecies level.

### 3.3. Mitochondrial COI and ITS-1 Gene Sequence Differences Analysis of C. japonica

**Figure 4:**
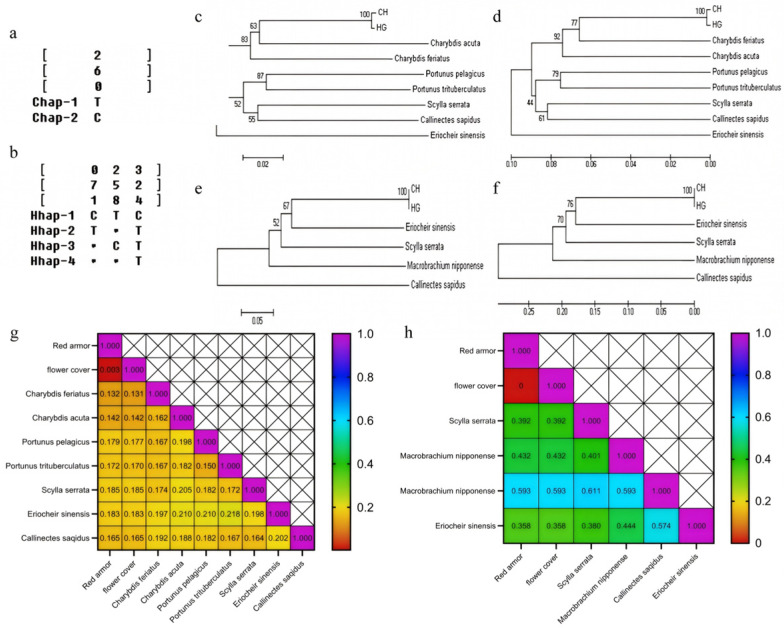
Mitochondrial COI and ITS-1 gene sequence differences analysis of two color morphs of *C. japonica*. The variation sites of COI sequences of different haplotypes of red morph (**a**) and green morph (**b**). Phylogenetic trees of N-J (**c**) and UPGMA (**d**) based on COI gene fragments. Phylogenetic trees of N-J (**e**) and UPGMA (**f**) based on ITS-1 gene fragments, CH represented red morph, and HG represented green morph. Genetic distances between *C. japonica* and peripheral species calculated based on COI (**g**) and ITS-1 (**h**) gene fragments.

The sequence results based on COI gene showed that the sequence identity between red and green morph groups was 98.96%. The length of gene fragments of red and green morph groups was 673 bp and 671 bp, respectively. The A, T, C, G contents in red and green morph groups were 26.3%, 37.1%, 18.4%, 18.1%, and 26.4%, 36.9%, 18.5%, 18.2%, respectively. In *C. japonica* samples, the total content of A and T was higher than that of C and G content, which is a characteristic feature observed in invertebrate mitochondrial DNA sequences. Furthermore, as shown in Figure 4, sequence alignment results revealed that six individuals in the red morph group exhibited two haplotypes (Chap-1 and Chap-2), with one individual showing a T-to-C transition at a single mutation site. Whereas, four haplotypes (Hhap 1, 2, 3, 4) were detected in six individuals of the green morph groups. In addition, three variable sites were detected in green morph group, including two single-variable sites and one parsimony-informative site. All three sites involved transitions between T and C. As observed in Figure 4g, the genetic distance between both groups was 0.003, while the genetic distance between them and peripheral species ranged from 0.131 to 0.185. The phylogenetic tree constructed by N-J and UPGMA methods based on COI gene fragments also presented same trend (Figure 4c,d). The red and green morph groups were initially clustered together and then clustered into different branches with the other two species of Charybdis. *Scylla serrata* was first clustered with *Callinectes sapidus*, and then formed a group with *Portunus* genus, which included *Portunus trituberculatus* and *Portunus pelagicus*, and finally clustered with *Eriocheir sinensis*.

The ITS-1 gene sequence analysis showed a high similarity (99.71%) between red and green morph groups, with gene fragment lengths of 360 bp and 359 bp, respectively. The average A, T, C, and G content in the red morph group was 23.7%, 23.4%, 23.1%, and 29.8%, respectively, while the average content of A, T, C, and G in the green morph groups was 23.9%, 23.4%, 23.1%, 29.8%, respectively. The sequence alignment showed that only one out of six individuals exhibited a 12 bp deletion at locus 205–216, whereas all other loci were identical across the samples. In green morph group, four haplotypes (HGhap 1, 2, 3, 4) were detected among six individuals. In addition, four variable sites were also observed, with two sites showing transitions between A and G, and the other two sites presented transitions between T and G. As shown in Figure 4h, the genetic distance between two groups was 0.000, while the genetic distance between them and peripheral species was ranged from 0.358 to 0.593. The phylogenetic tree constructed based on the ITS-1 gene showed that the red morph and green morph clustered together first, then sequentially clustered with the *Eriocheir sinensis*, *Scylla serrata*, *Macrobrachium nipponense*, and finally clustered with the *Callinectes sapidus*.

### 3.4. Differences in Muscle Quality Between Two Color Morphs of C. japonica

**Figure 5:**
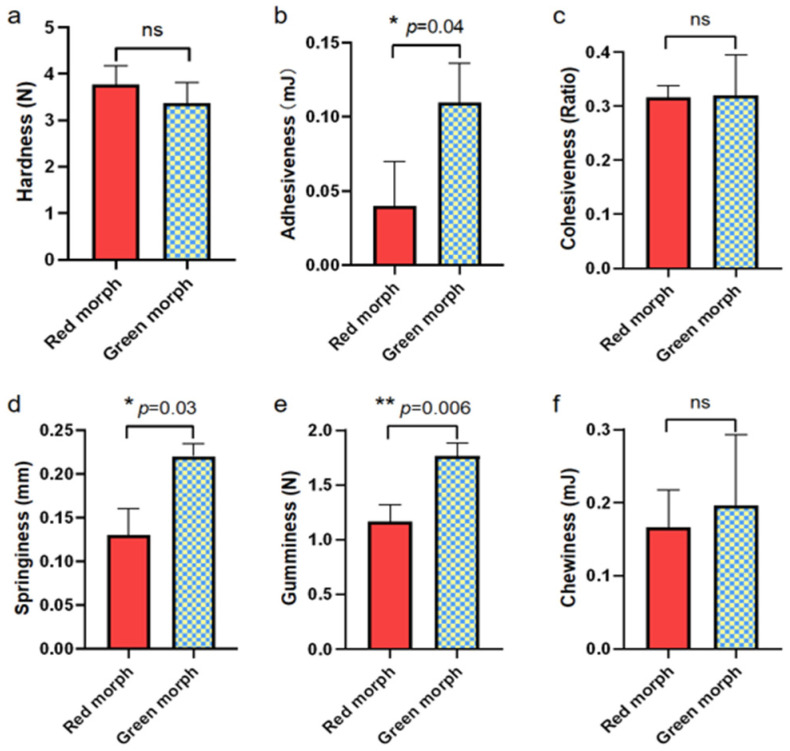
Comparison of muscle texture characteristics ((**a**): hardness, (**b**): adhesiveness, (**c**): cohesiveness, (**d**): springiness, (**e**): gumminess, (**f**): chewiness) between two color morphs of *Charybdis japonica*. Vertical bars represent the mean ± SD (*n* = 3). * means significant difference (*p* < 0.05), ** means highly significant difference (*p* < 0.01), ns means no significant difference (*p* > 0.05).

As shown in Figure 5, no significant differences were observed in the hardness, cohesiveness, and chewiness of red and green morph groups. However, adhesiveness (*p* = 0.04), springiness (*p* = 0.03), and gumminess (*p* = 0.006) in the green morph group were significantly higher compared to the red morph group. Moreover, no significant difference was observed in the muscle amino acid composition of both red and green morphs as observed in Table 5.

## 4. Discussion

The results of morphological and mitochondrial DNA analysis indicate that both red and green color morphs of *C. japonica* belong to the same species. Previously, Hidayani et al. reported significant morphometric variation in *Portunus* sp. from West Papua, Indonesia, highlighting substantial intraspecific diversity within the species [27]. Another study also revealed morphological differences among the four wild populations of *Portunus triuberbuculatus* in China. Certain morphological variations were observed among the populations, but no distinctions were found at the subspecies level [28]. In the present study, visual differences in appearance and body color were observed between both groups, but no significant morphological differences were detected between the female *C. japonica* from both groups. Among male *C. japonica*, significant differences were recorded in the ratios of CL/CW, BW/CW, and PW/CW. However, the coefficient of variation for these three parameters was below 1.28. According to the taxonomic classification criteria, the morphological difference between the two color morphs did not exceed the threshold for subspecies distinction in *C. japonica*. This suggests variations are likely due to differences in geographical locations rather than representing distinct subspecies [29]. The mitochondrial DNA (mtDNA) is widely utilized to investigate genetic diversity in various crustaceans species, including Chinese prawn (*Feneropenaeus chinensis*), mitten crab (*Eriocheir sensu stricto*), and red spotted swimming crab (*P. sanguinolentus*) [30,31,32]. In this study, the nucleotide homology of mitochondrial COI and ITS-1 gene fragments between red and green morphs was 98.96% and 99.71%, respectively. The comparison of these target gene sequences provides direct insight into the genetic relationship between the two morphs. In bacterial taxonomy, a sequence homology of 97% is often used as a threshold to determine whether two groups likely belong to the same species [33]. Based on this criterion, two color morphs of *C. japonica* should indeed belong to the same species. It is a common natural phenomenon for individuals or populations of the same species to exhibit variations in body color. Previously, a study on *P. trituberculatus* demonstrated that the COI gene sequence homology between its purple and green morphs in nature approaches 100%, confirming that they belong to the same species. This suggested that the purple morphs of *P. trituberculatus* have not undergone any subspecies differentiation [34]. In the present study, neither the mtDNA COI nor the ITS-1 gene sequences revealed any distinct differences that could be used for distinguishing the two body-color morphs of *C. japonica*. Furthermore, studies can be conducted to investigate conserved mtDNA sequences, such as the 16S rRNA gene, which is frequently employed to elucidate phylogenetic relationships among crustacean species [35]. However, the reasons behind the morphological differences between the red and green morphs of *C. japonica* are unclear and warrant further exploration. Certain factors, such as genetics, physiology, biochemistry, molecular biology, and environmental conditions, may be responsible for the different body colors of *C. japonica*. Moreover, a comprehensive understanding of the relationship between body color and other biological aspects of *C. japonica* is essential for identifying the mechanisms responsible for color variations within the same species.

The CIELAB color space, often referred to as Lab*, is a widely adopted digital method for assessing color characteristics in aquatic animals. In this study, the carapace of red morph group exhibited higher a* value and lower L* and b* values compared to the green morph group. The differences in body color also lead to distinct behavioral traits in both groups. Previous research has demonstrated that the courtship behavior of *C. japonica* is influenced by body color, with both red and green morph females exhibiting significantly higher mating rates with red morph males [36]. Additionally, a study on *Uca pugilator* revealed that color or brightness as visual signals can promote mate selection, rather than relying on chemical signals [37]. In aquatic environments, visual signals may propagate faster and cover greater distances compared to chemical signals. Furthermore, variations in Lab* values were also observed in the inner membranes of two body colors morphs of *C. japonica*. The inner membrane, as part of the exoskeleton in crustaceans, plays a critical role in determining body color, primarily due to the presence and distribution of pigments in it [38]. The inner membrane of red morph showed higher melanocyte aggregation and a greater density of erythrocytes compared to the green morph, which directly influenced their body colors. We speculate that these variations in body color may be influenced by environmental factors, which can affect the distribution and synthesis of pigment cells in crustaceans. Previous studies have also shown that *Uca pugilator* can alter its body color in response to the temperature and light intensity of the surrounding environment [39,40]. The natural occurrence of both *C. japonica* groups in different locations also exposed them to distinct environmental conditions. The red morph group is predominantly distributed in nearshore rocky substrates, while the green morph group inhabits deeper muddy substrates. This distributional difference exposes these two color morphs to varying environmental factors, potentially influencing their body color. Previous research suggested that the sensitivity of photoreceptors in *Haptosquilla trispinosa* was inversely proportional to water depth due to the absorption of long-wavelength light by seawater [41]. Another study indicated that short-wavelength light affects melanin production in peripheral tissues through the visual protein Opn5m [42]. However, in the natural environment, body color is influenced by both environmental factors and dietary pigments, making the situation more complex. Moreover, the type and concentration of dietary pigments exhibit significant impact on body color of crustaceans [43]. Therefore, future research should focus on exploring the mechanisms underlying the variations in body color of *C. japonica* by analyzing the effects of dietary pigment supplementation and identifying key environmental factors. Such studies will provide deeper insights into how various factors such as genetics, environment, and diet interact to influence the phenotypic diversity of this species.

Muscle texture is a comprehensive index that describes the physical and sensory characteristics of muscle tissue. Thus, muscle texture exhibits a direct impact on the consumer acceptance and market value of the product. Hardness of muscle is referred to as the minimum force required to deform a sample, directly reflecting the toughness of meat texture; Adhesiveness is the energy required for the probe to withdraw from the sample after a single compression, indicating the strength of intercellular binding forces in a particular meat sample. The greater intercellular binding forces results in higher adhesiveness; Cohesiveness reflects the ability of a meat sample to resist damage and maintain structural integrity through tight cellular connections; Chewiness represents the work (energy) required to masticate a solid sample into a swallowable state; Springiness is the ability of the sample to recover its original shape after the removal of the deforming force; Gumminess is a product of hardness x cohesiveness, used to reflects chewiness of muscle meat [44].

In this experiment, the muscle of green morph group exhibited higher levels of adhesiveness, springiness, and gumminess compared to the red morph group. Thus, muscles from the green morph group are likely to perceive softer and more compact while chewing. It is speculated that the distribution and content of carotenoids may have imposed a significant influence on the textural properties of muscle tissue in both groups of *C. japonica*. It was also reported that exposure to oxidative stress inhibits collagen production in fibroblasts, while reactive oxygen species (ROS) damage fibroblasts by interfering with collagen synthesis and extracellular matrix remodeling [45]. As antioxidants, carotenoids can effectively alleviate the adverse effects of ROS on muscle fibers. Previously, dietary lutein supplementation significantly improved muscle adhesiveness, cohesiveness, springiness, gumminess, and chewiness of southern catfish (*Silurus soldatovi meridionalis* Chen) [46]. In addition, a limited number of replicates for texture analysis may result in a lack of significant differences in texture analysis of muscle between the two color morphs. Thus, future studies should integrate novel methodologies such as image processing and acoustic analysis techniques with subjective evaluation by professionally trained panelists to comprehensively understand fish textural properties.

Crustacean muscle tissues contain a significant quantity of free amino acids, which contribute to the unique umami and sweetness of the meat. For instance, the levels of alanine (Ala) and glycine (Gly) are closely associated with sweetness, whereas glutamate (Glu) and aspartate (Asp) are strongly linked to umami flavor [47]. Although no significant difference was observed in the amino acid composition of muscles of both groups in the present study, this does not necessarily indicate that the overall flavor profile of both groups is identical. In this experiment, the limited sample size (*n* = 3) may be a key factor contributing to the insignificant results. Thus, future studies should also consider increasing the sample size. Additionally, more sensitive detection techniques or an expanded analysis scope may be considered to accurately detect and validate any potential differences in amino acid composition. Furthermore, a detailed analysis of volatile composition can also be considered to comprehensively investigate and compare the flavor differences between both groups of *C. japonica*.

There are still many limitations in this study, as we conducted sample collection only in the coastal waters of Dalian, which may restrict the generalizability of our conclusions. Due to the preliminary and exploratory nature of this research, a single sampling site was selected to control environmental variables, with a focus on genetic and morphological differences. We aim to propose an integrative framework to explain color variation in *C. japonica*, incorporating genetic, environmental, and dietary factors. According to the framework, genetic factors provide the foundation for body coloration, while environmental variables and diet modulate phenotypic expression through gene–environment interactions. To validate this overall framework, several follow-up studies need to be conducted. First, we will expend the sampling range and conduct cross-regional species comparisons to verify whether morphological differences are correlated with environmental factors. Second, to uncover the complex relationships among environment, gene expression, and phenotype, we will systematically measure the habitats of *C. japonica* and integrate nuclear gene markers, transcriptomic analysis, and functional validation of candidate genes. Finally, to comprehensively elucidate the genetic and physiological pathways underlying pigment variation, specific pigments such as carotenoids and biliverdin will be identified using HPLC or spectrophotometry, and association analyses incorporating environmental factors or gene expression data will further reveal the mechanisms underlying body color formation.

## 5. Conclusions

In this study, differences in the body color and muscle quality of two color morphs of *C. japonica* were compared based on their morphology and mitochondrial DNA. Both groups of *C. japonica* with different body colors were confirmed to belong to the same species. Significant differences in the textural characteristics were also observed in both color morphs of *C. japonica*, while their muscle amino acid composition was similar. Overall, this research contributes important practical implications for *C. japonica* aquaculture. First, body color variation can serve as a reference indicator for selective breeding, facilitating the development of high-value strains. Second, variations in the muscle textural properties provide a basis for optimizing processing of *C. japonica* for subsequent human consumption. Finally, this study confirms that both color morphs belong to the same species, preventing misclassification due to morphological differences and ensuring unified management of germplasm resources. This study also enhances the understanding of the biological basis of color variation in *C. japonica* and provides valuable insights for aquaculture practices and conservation strategies. Future studies should focus on investigating specific ecological and physiological mechanisms driving color variation in *C. japonica* for the comprehensive elucidation of key factors influencing its coloration and overall quality.

## Figures and Tables

**Table 1:** Species, accession number and length of outgroup species.

Species	Genebank Accession Number	Length of Fragments (bp)
*Charybdis feriatus*	EU284140 (COI)	709
*Charybdis acuta*	EU284143 (COI)	709
*Portunus pelagicus*	DQ889124 (COI)	657
*Portunus trituberculatus*	GU321231 (COI)	658
*Scylla serrata*	JN085429 (COI)	659
	AY181979 (ITS-1)	653
*Eriocheir sinensis*	HQ534047 (COI)	686
	AY181978 (ITS-1)	612
*Callinectes saqidus*	AY682078 (COI)	1534
	AY781436 (ITS-1)	1864
*Macrobrachium nipponense*	EU346851 (ITS-1)	1725

**Table 2:** The morphologic parameters of two color morphs of C. japonica.

Morphologic Parameters	♂ Red Morph (*n* = 32)	♂ Green Morph (*n* = 40)	♀ Red Morph (*n* = 33)	♀ Green Morph (*n* = 31)
CL	51.72 ± 4.54 a	47.96 ± 5.03 b	46.29 ± 4.54 b	44.31 ± 2.87 b
CW	77.91 ± 7.50 a	71.27 ± 8.25 b	68.84 ± 6.45 b	66.44 ± 4.39 b
CH	29.50 ± 2.98 a	28.57 ± 9.01 a	25.86 ± 2.39 a	25.82 ± 3.45 a
BW	111.57 ± 36.16 a	80.49 ± 29.07 b	66.21 ± 18.98 b	58.06 ± 10.77 b
FOMW	38.99 ± 2.46 a	36.41 ± 3.12 b	35.68 ± 3.19 b	34.38 ± 1.87 b
FFLC	26.93 ± 3.99 a	24.25 ± 3.58 b	19.01 ± 1.87 c	18.50 ± 1.65 c
MLC	30.40 ± 3.75 a	27.48 ± 4.08 b	22.47 ± 2.66 c	21.51 ± 1.57 c
PW	22.84 ± 3.84 a	17.59 ± 2.81 b	16.66 ± 2.73 b	15.53 ± 1.83 b
ST	0.71 ± 0.12 a	0.62 ± 0.09 b	0.63 ± 0.07 b	0.60 ± 0.07 b

Values presented as mean ± SE of samples. Values of each parameter in the same row with different superscripts are significantly different (*p* < 0.05). CL represents carapace length, CW represents carapace width, CH represents carapace height, BW represents body weight, FOMW represents first obital margin width, FFLC represents fixed finger length of claw, MLC represents meropodit length of the claw, PW represents pincer width, and ST represents shell thickness. ♂ represents male, and ♀ represents female.

**Table 3:** The morphologic parameters ratio statistics of two color morphs of *C. japonica*.

Morphologic Parameters Ratio	♂ Red Morph (*n* = 32)	♂ Green Morph (*n* = 40)	♀ Red Morph (*n* = 33)	♀ Green Morph (*n* = 31)
CL/CW	0.66 ± 0.01 ^b^	0.67 ± 0.02 ^a^	0.67 ± 0.01 ^a^	0.67 ± 0.01 ^a^
CH/CW	0.38 ± 0.01 ^a^	0.40 ± 0.10 ^a^	0.38 ± 0.01 ^a^	0.39 ± 0.05 ^a^
FOMW/CW	0.50 ± 0.02 ^a^	0.51 ± 0.02 ^a^	0.52 ± 0.01 ^a^	0.52 ± 0.01 ^a^
FFLC/CW	0.34 ± 0.02 ^a^	0.34 ± 0.02 ^a^	0.28 ± 0.01 ^b^	0.28 ± 0.01 ^b^
MLC/CW	0.39 ± 0.02 ^a^	0.39 ± 0.02 ^a^	0.33 ± 0.02 ^b^	0.32 ± 0.01 ^b^
PW/CW	0.29 ± 0.03 ^a^	0.25 ± 0.02 ^b^	0.24 ± 0.02 ^b^	0.23 ± 0.02 ^b^
BW/CW	1.40 ± 0.33 ^a^	1.10 ± 0.25 ^b^	0.95 ± 0.19 ^c^	0.87 ± 0.11 ^c^
ST/CW	0.01 ± 0.00 ^a^	0.01 ± 0.00 ^a^	0.01 ± 0.00 ^a^	0.01 ± 0.00 ^a^

Values presented as mean ± SE of samples. Values of each parameter in the same row with different superscripts are significantly different (*p* < 0.05). CL represents carapace length, CW represents carapace width, CH represents carapace height, BW represents body weight, FOMW represents first obital margin width, FFLC represents fixed finger length of claw, MLC represents meropodit length of the claw, PW represents pincer width, and ST represents shell thickness. ♂ represents male, and ♀ represents female.

**Table 4:** The morphologic parameters ratio of high variance between two color morphs of *C. japonica*.

Morphologic Parameters Ratio	♂ Red Morph	♂ Green Morph	Coefficient of Variation
CL/CW	0.66 ± 0.10	0.67 ± 0.02	0.37
BW/CW	1.40 ± 0.33	1.10 ± 0.25	0.52
PW/CW	0.29 ± 0.03	0.25 ± 0.02	1.02

Values presented as mean ± SE of samples. CL represents carapace length, CW represents carapace width, BW represents body weight, and PW represents pincer width. ♂ represents male, and ♀ represents female.

**Table 5:** The muscle amino acid composition between two color morphs of *C. japonica*.

Amino Acids (g/100 g Dry Matter)	Content		
	Red Morph	Green Morph	*p*-Value
Essential amino acid (EAA)			
Leucine	0.93 ± 0.23	0.89 ± 0.21	0.827
Isoleucine	0.53 ± 0.14	0.48 ± 0.12	0.662
Phenylalanine *	0.48 ± 0.08	0.47 ± 0.09	0.807
Lysine	0.88 ± 0.29	0.85 ± 0.28	0.905
Methionine	0.18 ± 0.11	0.21 ± 0.08	0.732
Threonine	0.52 ± 0.06	0.51 ± 0.05	0.847
Valine	0.59 ± 0.15	0.54 ± 0.14	0.695
Semi essential amino acid (SEAA)			
Histidine	0.26 ± 0.06	0.25 ± 0.07	0.841
Arginine	1.29 ± 0.35	1.04 ± 0.11	0.307
Non-essential amino acid (NEAA)			
Glutamic Acid *	1.60 ± 0.46	1.50 ± 0.50	0.807
Aspartic Acid *	0.87 ± 0.31	0.84 ± 0.31	0.929
Glycine	1.08 ± 0.24	1.03 ± 0.18	0.754
Alanine *	0.91 ± 0.22	0.83 ± 0.21	0.660
Serine	0.37 ± 0.10	0.36 ± 0.10	0.886
Proline	0.74 ± 0.07	0.78 ± 0.04	0.497
Tyrosine *	0.43 ± 0.05	0.45 ± 0.13	0.799
∑AA	11.66 ± 2.84	11.01 ± 2.52	0.779
∑EAA	4.11 ± 1.03	3.94 ± 0.96	0.845
∑DAA	4.29 ± 1.11	4.08 ± 1.22	0.839

* represents delicious amino acid (DAA). Values are presented as the means ± SD (*n* = 3).

## Data Availability

The raw data supporting the conclusions of this article will be made available by the authors on request.

## Supplementary Materials

The following supporting information can be downloaded at: https://www.mdpi.com/article/10.3390/foods14142516/s1, Figure S1: Body Weight Distribution of two color morphs of *C. japonica*.
