# Supplementary figures and images for: Genetic, Diversity, and Muscle Quality Among Red and Green Color Morphs of Asian Swimming Crab (Charybdis japonica): Implications for Accurate Species Recognition and Sustainable Management
Source: Foods. 2025 Jul 18;14(14):2516. doi: 10.3390/foods14142516 (PMC12294135; doi:10.3390/foods14142516)

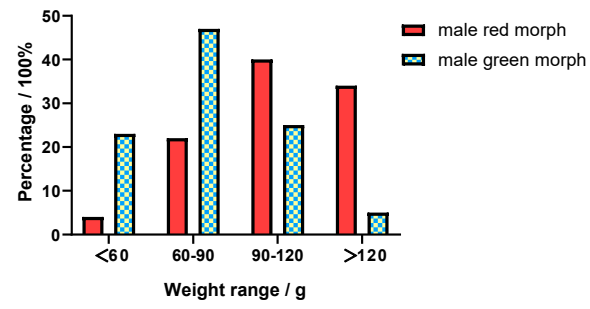

Figure S1. Body Weight Distribution of two color morphs of *C. japonica*

Supplement: Supplementary file 1 [file foods-14-02516-s001.zip › foods-3688081-supplementary.pdf]
